# Self-Toughened Epoxy Resin via Hybridization of Structural Isomeric Curing Agents

**DOI:** 10.3390/polym17050695

**Published:** 2025-03-05

**Authors:** Woong Kwon, Jiyeon Cheon, Hei Je Jeong, Jong Sung Won, Byeong-Joo Kim, Man Young Lee, Seung Geol Lee, Euigyung Jeong

**Affiliations:** 1 Department of Textile System Engineering, Kyungpook National University, Daegu 41566, Republic of Korea; kwoong7242@knu.ac.kr (W.K.); ziyun32@naver.com (J.C.); 2 Department of Materials Science and Engineering, Ulsan National Institute of Science and Technology, Ulsan 44919, Republic of Korea; vkdnjchl@gmail.com; 3 Defense Materials & Energy Technology Center, Agency for Defense Development, Yuseong P.O. Box 35, Daejeon 34060, Republic of Korea; jswon@add.re.kr (J.S.W.); dhfldhs727@naver.com (B.-J.K.); manyounglee@add.re.kr (M.Y.L.)

**Keywords:** epoxy resin, fracture toughness, diaminodiphenyl sulfone, curing agent, structural isomers

## Abstract

Fracture toughness is a key property of epoxy resins with a high glass transition temperature (T_g_), used in carbon fiber/epoxy composites for aerospace applications. Conventional toughening methods rely on adding toughening agents, often compromising the processibility and thermal stability. This study introduces a simple self-toughening approach that enhances the fracture toughness without sacrificing other properties by controlling the cured epoxy network structure. Tetraglycidyl 4,4′-diaminodiphenylmethane (TGDDM) epoxy resin was cured using mixtures of structural isomeric curing agents, 3,3′- and 4,4′-diaminodiphenyl sulfone (3,3′- and 4,4′-DDS), at ratios of 7:3, 5:5, and 3:7. The optimal 7:3 ratio produced a resin with 30% higher fracture toughness compared to TGDDM/3,3′-DDS and 100% higher than the TGDDM/4,4′-DDS system. The T_g_ of the self-toughened resin ranged from 241 to 266 °C, which was intermediate between the T_g_ values of the TGDDM/3,3′-DDS and TGDDM/4,4′-DDS systems. This improvement is attributed to the higher crosslink density and reduced free volume of the epoxy network. These findings demonstrate that simply mixing isomeric curing agents enables self-toughening, providing a practical and efficient strategy to enhance the performance of high-T_g_ epoxy resins in advanced composite applications.

## 1. Introduction

Epoxy resins are widely utilized across various industries due to their excellent thermal and mechanical properties, dimensional stability, and adhesive characteristics [1,2,3]. In particular, fiber-reinforced polymer composites (FRPs) with epoxy resin matrices exhibit outstanding specific strength and thermal stability, positioning them as a promising alternative to metals for high-performance aerospace structural applications [4,5,6,7]. For such applications, epoxy resins must exhibit exceptional thermal and mechanical properties, particularly a high glass transition temperature (T_g_), which is achieved through highly crosslinked network structures formed using multifunctional epoxy resins such as triglycidyl *p*-aminophenol (TGPAP) and tetraglycidyl diaminodiphenylmethane (TGDDM) in combination with aromatic amine curing agents [8,9]. However, while these highly crosslinked structures ensure excellent thermal performance, they also increase the brittleness, reducing the toughness and compromising the durability [10]. Consequently, significant efforts have focused on optimizing the balance between strength and toughness to ensure their suitability for advanced aerospace materials [11,12,13,14,15,16,17,18,19,20,21,22,23,24].

The most common strategies to enhance the fracture toughness of high-T_g_ epoxy resins involve introducing secondary phases, such as rubber, inorganic particles, or thermoplastic resins [11,12,13,14,15,16,17,18,19,20]. Although these methods effectively improve the toughness, they often introduce challenges. For instance, rubber addition compromises the thermal properties due to its low T_g_ [11,12,13,14], while inorganic particles may agglomerate, leading to poor dispersion, stress concentration points, and eventual mechanical degradation [15,16,17]. Thermoplastic resins improve the fracture toughness without significantly affecting the thermal or mechanical properties, but achieving optimal toughness often requires high concentrations of thermoplastics, which increase the resin viscosity and reduce the processability [18,19,20].

An alternative approach is modifying the crosslinking density. For example, Zubeldia et al. demonstrated that varying the stoichiometric ratios in diglycidyl ether of bisphenol A (DGEBA) cured with 4,4′-diaminodiphenyl sulfone (DDS) improved the fracture toughness by up to 91% [22]. Similarly, Levita et al. showed that reducing the crosslinking density by inducing incomplete curing in DGEBA/DDS systems increased the fracture toughness by 70% [23]. However, such methods often involve trade-offs, including reduced thermal or mechanical properties and increased manufacturing complexity. This underscores the need for effective toughening strategies that minimize these drawbacks.

This study introduces a facile method to enhance the fracture toughness of high-T_g_ epoxy resins by hybridizing structural isomeric curing agents. Previous research has demonstrated that the position of the amine group in DDS significantly influences the mechanical properties. For example, Kwon et al. reported that TGPAP cured with 3,3′-DDS exhibited 44% higher fracture toughness than TGPAP cured with 4,4′-DDS [24]. Sahagun et al. showed that differences in the reactivity of 3,3′- and 4,4′-DDS resulted in variations in the crosslinking network structure [25]. Additionally, Roderick et al. reported that when the isomers of the trifunctional epoxy resin (triglycidly *m*-aminophenol and TGPAP) were cured using DDS isomers, 3,3′-DDS and 4,4′-DDS, respectively, the differences in the isomeric structures led to variations in the packing efficiency, intermolecular interactions, and crosslink density of the cured resins, leading to changes in the packing and cohesive energy within the epoxy network. These changes alter the molecular mobility, inducing the internal anti-plasticization of the network, which in turn impacts the fracture toughness of the epoxy resin. As a result, the resin cured with 3,3′-DDS exhibited 11% higher fracture toughness compared to the resin cured with 4,4′-DDS [26]. Building on these findings, this study explores a self-toughening approach for high-T_g_ epoxy resins by modifying the crosslinking structure of epoxy through the hybridization of 3,3′- and 4,4′-DDS with various ratios. Advanced aerospace materials require a T_g_ of over 220 °C [27]. The epoxy system utilizing TGDDM and DDS is recognized as a high-T_g_ epoxy resin, demonstrating a T_g_ exceeding 240 °C [28,29]. Therefore, in this study, a mixture of 3,3′-DDS and 4,4′-DDS was selected as a curing agent for TGDDM, which is commonly used in aerospace composite materials, to improve the fracture toughness. The resulting cured epoxy resins were evaluated for their fracture toughness, tensile properties, and thermal properties, with analyses of the free volume and crosslinking density conducted to elucidate the effects of isomeric curing agent blends on the epoxy network structure.

## 2. Materials and Methods

### 2.1. Materials

TGDDM (95%) was purchased from Sigma-Aldrich (St. Louis, MO, USA). The curing agents 3,3′-DDS (98%) and 4,4′-DDS (98%) were obtained from the Tokyo Chemical Industry (Tokyo, Japan). Methanol (99%), ethanol (99%), propanol (99%), and butanol (99%) were purchased from Daejung Chemical Co., Ltd. (Ansan, Republic of Korea).

### 2.2. Preparation of Epoxy Thermosets

TGDDM was mixed with 33 phr of DDS under stirring at 80 °C for 2 h. The mixture was then degassed at 80 °C for 10 min in a rotary evaporator. Following this, the mixture was cured at 150 °C for 2 h, 180 °C for 1 h, and 210 °C for 2 h. The resulting epoxy thermosets were labeled based on the mixing ratio of 3,3′- and 4,4′-DDS, as summarized in Table 1.

### 2.3. Solvent Uptake Measurements

Approximately 400 mg of each epoxy sample was dried in a vacuum oven at 100 °C for 24 h before solvent uptake measurements. The samples were placed in 25 mL vials containing 20 mL of solvent. Four solvents with varying van der Waals volumes—methanol, ethanol, propanol, and butanol—were used. The samples were stored at 25 °C for 10 days, with their surfaces wiped clean with a cotton cloth after the storage period. The weight of each specimen was measured, and the solvent absorption was calculated based on the weight change [30].

### 2.4. Characterization of Prepared Samples

The curing behavior of the epoxy samples was analyzed using differential scanning calorimetry (DSC, Q2000, TA Instruments, New Castle, DE, USA). The DSC instrument was purged with nitrogen at a flow rate of 50 mL/min. Approximately 10–15 mg of the TGDDM/DDS mixtures was heated in hermetically sealed aluminum pans, and an empty aluminum hermetic pan served as the reference. Dynamic DSC analysis was performed with heating rates of 2, 5, 10, and 20 °C/min from 25 to 300 °C. Isothermal DSC analysis was conducted at 160, 180, 200, and 220 °C for 2 h. The Fourier transform infrared (FT-IR) spectra of the prepared samples were obtained using an FT-IR spectrophotometer (Nicolet iS5, Thermo Fisher Scientific, Waltham, MA, USA) equipped with an attenuated total reflection accessory (iD7 ATR, Thermo Fisher Scientific, Waltham, MA, USA). The thermomechanical properties were evaluated using dynamic mechanical analysis (DMA, Discovery DMA850, TA Instruments, New Castle, DE, USA) under tension mode with a heating rate of 2 °C/min from 25 to 300 °C and a frequency of 1 Hz under nitrogen. The DMA specimen dimensions were 40.0 × 10.0 × 2.0 mm^3^, the applied force was 10 N, and the amplitude was 20 μm. The tensile properties and fracture toughness were measured using a universal testing machine (UTM, AG-5kNx, Shimadzu, Kyoto, Japan). The tensile tests followed the ASTM-D638 standard, with a crosshead speed of 2 mm/min. Fracture toughness was evaluated according to the ASTM-D5045 standard using single-edge-notched bend specimens and a three-point bending rig at a crosshead speed of 1 mm/min. After the fracture toughness test, the fracture surfaces at the locations near the crack tip were observed using a field emission scanning electron microscope (FE-SEM, SU8220, Hitachi, Tokyo, Japan) at an accelerating voltage of 5 kV. All samples were coated with platinum for 60 s to enhance the conductivity before analysis.

## 3. Results and Discussion

### 3.1. Curing Behaviors of the Prepared Samples

**Figure 1:**
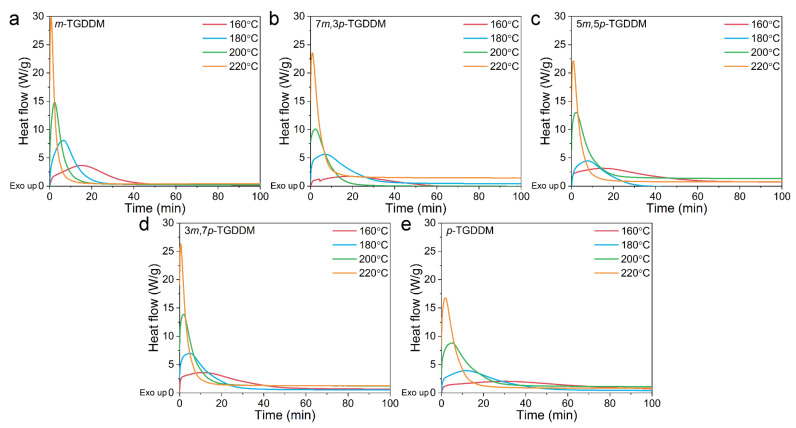
Heat flow from isothermal DSC of (**a**) *m*-TGDDM, (**b**) 7*m*,3*p*-TGDDM, (**c**) 5*m*,5*p*-TGDDM, (**d**) 3*m*,7*p*-TGDDM, and (**e**) *p*-TGDDM.

The isothermal DSC curves of the epoxy resins cured with different ratios of 3,3′- and 4,4′-DDS are presented in Figure 1. A comparison of the DSC curves for the epoxy resins cured individually with 3,3′-DDS (*m*-TGDDM) and 4,4′-DDS (*p*-TGDDM) reveals that the exothermic peak of *p*-TGDDM was broader and occurred at a slower rate across all tested temperatures compared to *m*-TGDDM. This suggests that the curing reaction for *p*-TGDDM proceeds more gradually.

For the epoxy resins cured with a combination of 3,3′- and 4,4′-DDS, the exothermic peak shifted to longer reaction times as the proportion of 4,4′-DDS increased from 30% to 70%. This indicates a delay in the curing reaction with higher 4,4′-DDS content, likely due to differences in reactivity between the two isomeric curing agents.

To quantitatively assess the curing behavior, the degree of conversion as a function of time was calculated using Equation (1) [31,32]:

(1) $$\alpha =\frac{∆H_{t}}{∆H_{R}}\times 100\;(\%),$$

where α is the degree of conversion, Δ*H_t_* is the cumulative heat released up to time *t*, and Δ*H_R_* is the total heat release.

**Figure 2:**
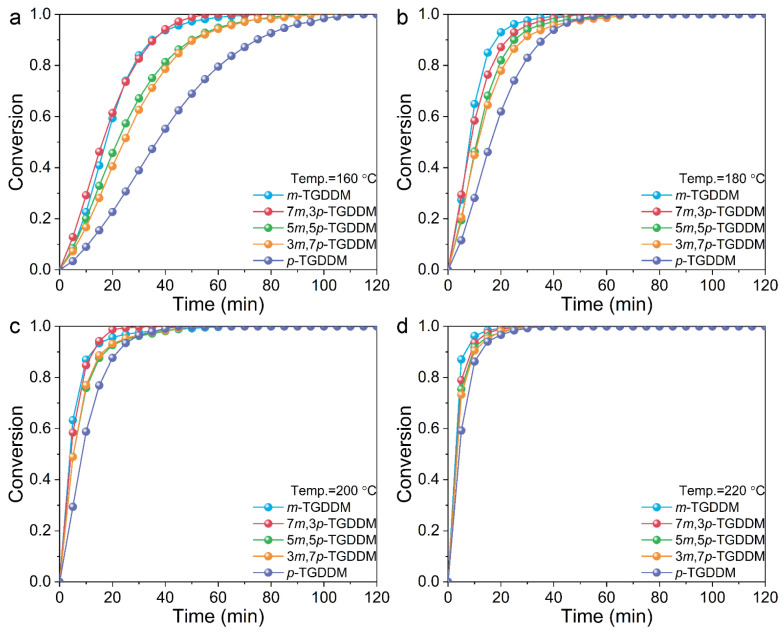
Degree of conversion as a function of time for the prepared epoxy samples at (**a**) 160, (**b**) 180, (**c**) 200, and (**d**) 220 °C.

As shown in Figure 2, the degree of conversion for all epoxy samples increased rapidly during the initial stages of the curing reaction. Higher curing temperatures significantly accelerated the conversion rates, enabling all samples to reach full conversion (100%) within a short period. However, in TGDDM systems cured with mixed curing agents of 3,3′- and 4,4′-DDS, the degree of conversion decreased progressively as the proportion of 4,4′-DDS increased. This trend highlights the influence of the curing agent composition on the reaction kinetics.

To further explore the effects of isomeric curing agents on the curing behavior of epoxy resins, the activation energy of the systems was analyzed using dynamic DSC (Figure S1). The curing reaction between epoxy and amine, which progresses through multiple stages, is very complicated. DSC was employed to assess the curing behavior, based on the assumption that the heat flow is proportional to the rate of the reaction between the epoxy and amine (Equation (2)) [33].

(2) $$\frac{d_{\alpha }}{d_{t}}=k\left(T\right)f(\alpha )$$

where *k*(*T*) is the reaction rate constant, α is the degree of conversion, and *f*(α) is a function of α that varies according to the reaction mechanism.

The reaction rate constant *k*(*T*) is defined by the Arrhenius equation, as presented in Equation (3):

(3) $$k\left(T\right)=Aexp(-\frac{E_{a}}{RT})$$

where *A* is the pre-exponential factor, *E_a_* is the activation energy, *R* is the universal gas constant, and *T* is the absolute temperature.

The Kissinger method calculates the activation energy of non-isothermal reactions based on the Arrhenius equation and is extensively used due to its simplicity in determining the activation energy. This method fundamentally assumes that the reaction rate is maximized at the peak temperature, as expressed in the following Equation (4) [34]:

(4) $$\ln\left(\frac{\theta }{T_{p}^{2}}\right)=ln(\frac{AR}{E_{a}})-\frac{E_{a}}{RT_{p}}$$

where *θ* is the heating rate and *T_p_* is the exothermic peak temperature.

ln(*θ/T_p_^2^*) exhibits a linear relationship with 1/*T_p_*, and the activation energy is determined by calculating the slope of the linear plot obtained by plotting ln(*θ/T_p_^2^*) vs. 1/*T_p_*.

The Flynn–Wall–Ozawa method is also employed to calculate the activation energy of non-isothermal reactions. This method assumes that both the activation energy and the pre-exponential factor are functions that vary with the degree of curing, as expressed in Equation (5) [35].

(5) $$\ln\left(\theta \right)=const.-1.052\frac{E_{a}}{RT_{p}}$$

A linear relationship exists between ln(*θ*) and 1/*T_p_*, and the activation energy is determined from the slope of the linear plot obtained by plotting these variables. These methods provide insights into the energy barrier associated with the curing reaction.

**Figure 3:**
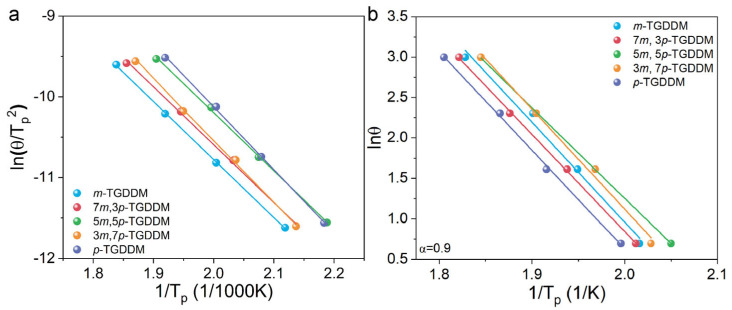
Fitted activation energy values obtained using the (**a**) Kissinger method and (**b**) Flynn–Wall–Ozawa method (α = 0.9).

As shown in Figure 3, the activation energies calculated using both the Kissinger and Flynn–Wall–Ozawa methods exhibited strong linear correlation coefficients (R^2^ > 0.99), confirming the reliability of the data. The Flynn–Wall–Ozawa method (average) yielded activation energies of 75.9 kJ/mol for *m*-TGDDM and 89.2 kJ/mol for *p*-TGDDM, while the Kissinger method produced values of 64.8 and 66.6 kJ/mol, respectively (Table 2). These results indicate that curing with 3,3′-DDS (*m*-TGDDM) required lower activation energy compared to 4,4′-DDS (*p*-TGDDM). The activation energy for *p*-TGDDM, calculated using the Flynn–Wall–Ozawa method (Figures S2 and S3), showed a decrease from 107.5 kJ/mol to 81.2 kJ/mol as the degree of conversion (α) increased from 0.1 to 0.4. Subsequently, as the conversion rate further increased from 0.5 to 1.0, the activation energy increased from 83.2 kJ/mol to 122.7 kJ/mol. In contrast, *m*-TGDDM exhibited a lower activation energy at the initial degree of conversion compared to *p*-TGDDM, and the activation energy increased as the degree of conversion rose. The decrease in the activation energy of *p*-TGDDM as the degree of conversion increased from 0.1 to 0.4 can be attributed to the autocatalytic effect caused by the formation of -OH groups from the reaction between primary amines and epoxy [36]. Furthermore, the increase in the activation energy when the degree of conversion exceeds 0.5 is due to the increase in the viscosity of the epoxy, which restricts the movement of polymer chains [36]. For systems using mixed curing agents, the activation energies ranged from 64.8 to 66.6 kJ/mol (Kissinger) and 75.9 to 89.2 kJ/mol (Flynn–Wall–Ozawa, average), with no clear trend observed between the mixing ratios and activation energies. Nevertheless, both methods consistently showed the lowest activation energy when the 3,3′-DDS content was 70% and the highest when it was 30%.

The differences in the curing behavior between 3,3′-DDS, 4,4′-DDS, and their mixtures can be attributed to the distinct reactivities of these isomers. In 4,4′-DDS, the sulfone group exerts a strong electron-withdrawing effect, delocalizing the nitrogen’s lone pair of electrons and reducing its nucleophilicity. In contrast, the amine groups in 3,3′-DDS retain higher nucleophilicity due to their positional arrangement, which prevents such delocalization [37]. When 3,3′- and 4,4′-DDS are used together, their differences in reactivity lead to more complex curing reactions, further influencing the overall curing behavior and activation energy profiles.

Furthermore, the chemical structural changes of the epoxy resin and curing agent mixture before and after curing were confirmed using FT-IR spectra (Figure S4). In the FT-IR spectra of the uncured TGDDM and DDS mixture, the peaks at 3460 and 3360 cm^−1^ were attributed to the NH_2_ stretching vibration and the peak at 900 cm^−1^ was attributed to the C-O stretching vibration in the epoxide group. Both the NH_2_ peak and the epoxide group peak disappeared in the FT-IR spectra of the epoxy resins, confirming that the curing reaction was successfully completed.

### 3.2. Mechanical and Thermal Properties of the Prepared Samples

**Figure 4:**
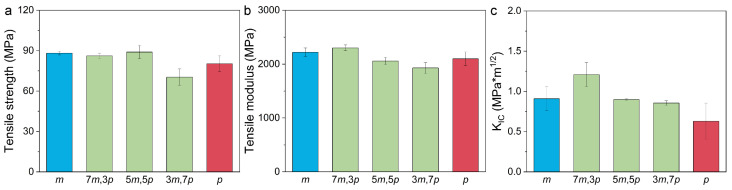
Mechanical properties of the prepared epoxy samples: (**a**) tensile strength, (**b**) tensile modulus, and (**c**) fracture toughness.

The mechanical properties of the epoxy resins cured with varying ratios of 3,3′- and 4,4′-DDS were evaluated, as shown in Figure 4. A comparison of the tensile properties revealed that TGDDM cured with 3,3′-DDS (*m*-TGDDM) exhibited tensile strength of 88 MPa and a tensile modulus of 2221 MPa. In contrast, TGDDM cured with 4,4′-DDS (*p*-TGDDM) displayed lower values, with tensile strength of 80 MPa and a tensile modulus of 2100 MPa, representing reductions of 9% and 5%, respectively. These results suggest that the mechanical properties of TGDDM are enhanced when cured with 3,3′-DDS compared to 4,4′-DDS.

The fracture toughness of *m*-TGDDM was measured at 0.9 MPa × m^1/2^, which is 50% higher than the 0.6 MPa × m^1/2^ observed for *p*-TGDDM. This can be attributed to the denser molecular chain packing associated with 3,3′-DDS, as reported in previous studies on difunctional epoxy resins cured with DDS isomers [38]. Denser chain packing reduces the free volume, increases the density, and improves the mechanical properties. This effect was similarly observed in the TGDDM/DDS system, where the structural characteristics of 3,3′-DDS enhanced the mechanical performance of the cured resin.

For TGDDM cured with mixtures of 3,3′- and 4,4′-DDS, the mechanical properties varied depending on the mixing ratio of the curing agents. The tensile strength was comparable to that of *m*-TGDDM, measuring 86 and 89 MPa for 7*m*,3*p*- and 5*m*,5*p*-TGDDM, respectively. However, it decreased by 20%, reaching 70 MPa, for 3*m*,7*p*-TGDDM. The tensile modulus decreased from 2303 to 1930 MPa as the proportion of 4,4′-DDS increased, with the highest value observed in 7*m*,3*p*-TGDDM.

The fracture toughness measurements also revealed significant variations. The fracture toughness of 5*m,*5*p*- and 3*m,*7*p*-TGDDM was 0.9 MPa × m^1/2^, similar to *m*-TGDDM. However, the highest fracture toughness of 1.2 MPa × m^1/2^ was observed for 7*m,*3*p*-TGDDM, representing improvements of 30% and 100% compared to *m*-TGDDM and *p*-TGDDM, respectively. Previous studies have demonstrated that the introduction of a secondary phase into the TGDDM/DDS system enhances the fracture toughness [39,40,41,42,43]. Specifically, the application of the thermoplastic toughening agent PES resulted in a 14–80% improvement in fracture toughness [38,39], the incorporation of polyetherimide yielded a 19–57% enhancement [40,41], and the use of amino-bearing phenyl silicone resins led to a 6–45% increase [42]. Although differences in the curing cycles between this study and previous studies limit direct quantitative comparisons, the toughening effects observed in this study are comparable to those reported in the literature. Moreover, because the toughening effect was achieved through the simple mixing of curing agents, the process is both straightforward and cost-effective, indicating promising potential for future industrial applications. The improvements in the tensile modulus and fracture toughness in samples with mixed curing agents can be attributed to differences in curing behavior and variations in the crosslinking structure caused by the distinct reactivities of 3,3′- and 4,4′-DDS.

These findings highlight the effectiveness of self-toughening achieved through the hybridization of structural isomeric curing agents. This approach enables the optimization of the mechanical properties without requiring additional toughening agents. A further discussion of these results is provided in Section 3.3.

**Figure 5:**
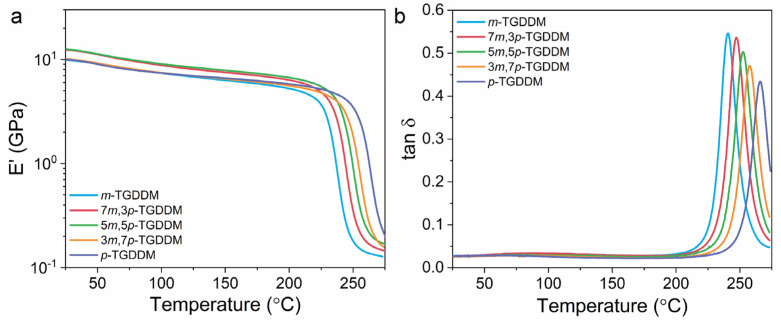
(**a**) Storage modulus and (**b**) tan δ curves of the prepared epoxy samples.

DMA was conducted to examine the thermal property variations of the epoxy resins based on the mixing ratio of 3,3′- and 4,4′-DDS. The results are presented in Figure 5 and Table 3. When either 3,3′-DDS or 4,4′-DDS was used individually as the curing agent, the storage modulus of the epoxy resin was approximately 10 GPa. However, when the curing agents were mixed, the storage modulus varied with the mixing ratio. Specifically, when 3,3′- and 4,4′-DDS were combined in ratios of 7:3 and 5:5 (7*m*,3*p*- and 5*m*,5*p*-TGDDM), the storage modulus increased to 12.5 GPa, representing an approximately 25% increase compared to using a single curing agent.

Above the T_g_, the storage modulus of the epoxy resin correlates with the crosslink density, which can be calculated using Equation (6) [44].

(6) $$\nu_{e}=\frac{E_{e}^{\prime }}{3RT}$$

where *ν_e_* is the crosslink density, *E*^′^_*e*_ is the storage modulus, *R* is the ideal gas constant, and T is the temperature at which the storage modulus is measured.

The crosslink densities of TGDDM cured with different ratios of 3,3′- and 4,4′-DDS as curing agents were calculated based on the storage modulus (Equation (6)) and are summarized in Table 3. The crosslink density of *m*-TGDDM was determined to be 9.28 × 10^3^ mol/m^3^, while, for *p*-TGDDM, it was 8.77 × 10^3^ mol/m^3^, indicating a 6% higher crosslink density when 3,3′-DDS was used as the curing agent. Additionally, the crosslink densities of 7*m*,3*p*- and 5*m*,5*p*-TGDDM were 10.8 × 10^3^ mol/m^3^ and 10.1 × 10^3^ mol/m^3^, respectively, which were up to 23% higher than those obtained using a single curing agent. These results demonstrate that using mixed curing agents produces a distinct crosslinking structure, leading to higher crosslink densities compared to single-agent systems.

The T_g_ of *m*-TGDDM was 241 °C, while, for *p*-TGDDM, it was 266 °C, indicating that 4,4′-DDS results in a higher T_g_. This difference is attributed to variations in the free volume and ring-flip behavior of the cured epoxy resin, which are directly influenced by the isomerism of the curing agents [38]. The phenyl ring in 4,4′-DDS undergoes flipping without requiring long-range molecular motion, whereas the molecular structure of 3,3′-DDS restricts such ring-flip behavior. The flipping of the phenyl ring absorbs thermal energy and impedes the movement of other molecular segments, which contributes to the higher T_g_ observed in TGDDM cured with 4,4′-DDS (*p*-TGDDM). In contrast, the restricted ring flip in 3,3′-DDS leads to a lower T_g_. For TGDDM cured with mixtures of 3,3′- and 4,4′-DDS, the T_g_ increased linearly with the proportion of 4,4′-DDS. This linear trend follows the mixing rule, indicating that the T_g_ of the epoxy resin is primarily governed by the ring-flip behavior, which is influenced by the isomerism of the curing agents.

### 3.3. Mechanism of Property Changes in Epoxy Samples

**Figure 6:**
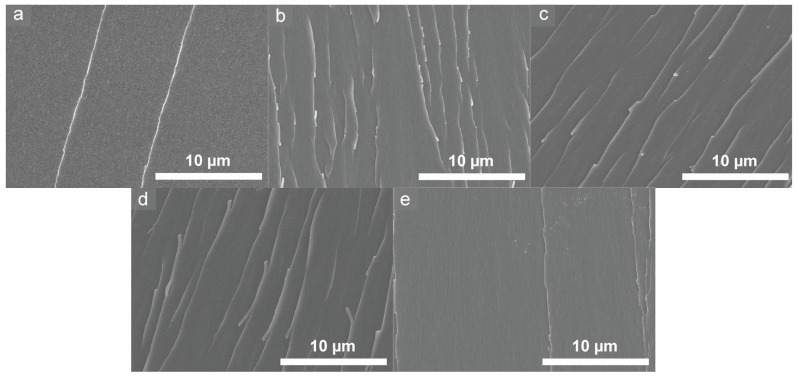
Fracture surfaces of the prepared epoxy samples: (**a**) *m*-TGDDM, (**b**) 7*m*,3*p*-TGDDM, (**c**) 5*m*,5*p*-TGDDM, (**d**) 3*m*,7*p*-TGDDM, and (**e**) *p*-TGDDM.

The fractured surfaces of the epoxy resins cured with varying mixing ratios of 3,3′- and 4,4′-DDS were analyzed using SEM to examine the impact of the curing agent composition on the fracture behavior. As shown in Figure 6, no significance difference in the energy dissipation mechanism was observed because there was no toughening agent added. However, the fractured surfaces themselves exhibited different shapes. The fractured surfaces of *m*- and *p*-TGDDM displayed smooth, clean features, which are characteristic of typical crack propagation in epoxy resins [45,46,47]. In contrast, the fracture surfaces of TGDDM cured with mixed curing agents also appeared smooth but exhibited denser, river-like patterns. These patterns suggest increased energy dissipation during fracture, indicating enhanced crack resistance. As discussed in Section 3.2, the mixing of curing agents with differing reactivities alters the crosslinking network, which improves the resistance to mechanical deformation, as reflected in the fracture morphology.

**Figure 7:**
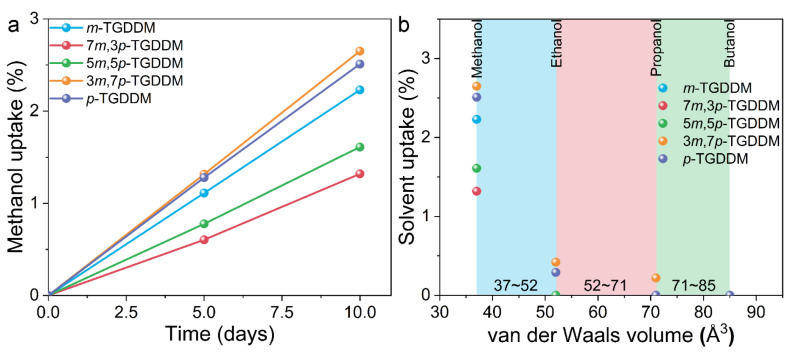
(**a**) Methanol uptake curves and (**b**) uptake data of various solvents for the prepared epoxy samples.

The solvent uptake of the epoxy resins was measured using methanol, ethanol, propanol, and butanol, with respective van der Waals volumes of 37, 52, 71, and 85 Å^3^ (Figure 7). Methanol uptake showed that *m*-TGDDM exhibited lower absorption than *p*-TGDDM, indicating a smaller free volume for *m*-TGDDM. For TGDDM cured with mixed curing agents, the methanol uptake in 7*m*,3*p*- and 5*m*,5*p*-TGDDM was lower than in *m*- and *p*-TGDDM. This suggests that the mixed curing agents altered the crosslinking structure and reduced the free volume. While methanol uptake occurred in all epoxy samples, solvents with larger van der Waals volumes (ethanol, propanol, and butanol) were absorbed only by specific samples, with significantly lower uptake observed overall (Figure 7b). For example, the ethanol uptake was 4% in *p*-TGDDM and 2% in 3*m*,7*p*-TGDDM, with no uptake observed in other samples. Notably, 3*m*,7*p*-TGDDM absorbed 0.6% propanol but did not absorb butanol, suggesting that its free volume size falls between 71 and 85 Å^3^. Based on these measurements, the estimated free volume sizes are as follows: *m*-TGDDM (37–52 Å^3^), 5*m*,5*p*-TGDDM (37–52 Å^3^), 7*m*,3*p*-TGDDM (37–52 Å^3^), *p*-TGDDM (52–71 Å^3^), and 3*m*,7*p*-TGDDM (71–85 Å^3^). Among these, 7*m*,3*p*-TGDDM, which exhibited the lowest methanol uptake, is expected to have the smallest free volume size.

**Figure 8:**
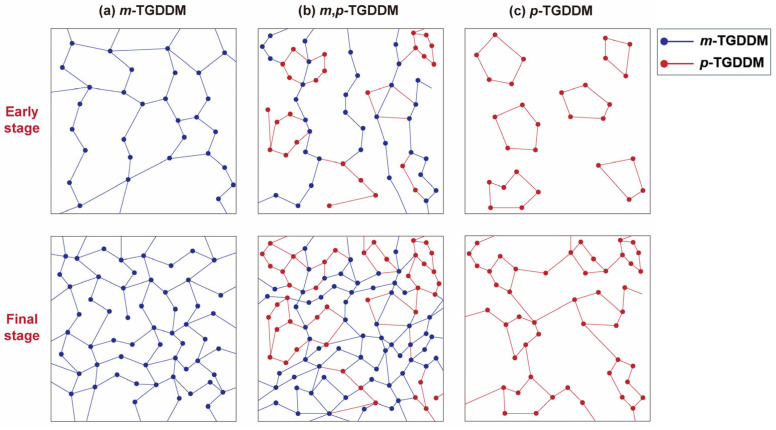
Schematic representation of crosslink structure growth in epoxy resins based on different types of curing agents: (**a**) *m*-TGDDM, (**b**) *m*,*p*-TGDDM, and (**c**) *p*-TGDDM. The blue dot with a blue line and the red dot with a red line represents *m*-TGDDM and *p*-TGDDM, respectively.

Figure 8 illustrates the network formation process of TGDDM, based on the type of curing agent used. In *m*-TGDDM (Figure 8a), the high reactivity of 3,3′-DDS leads to rapid, sequential reactions, with primary amines reacting first, followed by secondary amines [25]. This preferential reaction of primary amines promotes linear network growth (upper left), resulting in a uniform and dense crosslinked structure with a smaller free volume (lower left). In *p*-TGDDM (Figure 8c), the simultaneous reaction of both primary and secondary amines creates heterogeneous crosslinked domains (upper right), resulting in a larger free volume. For TGDDM cured with a mixture of 3,3′- and 4,4′-DDS (Figure 8b), regions of uniform network growth coexist with domain-driven growth (upper middle), leading to a more complex and denser crosslinked structure (lower middle). This denser network reduces the free volume, inducing the internal anti-plasticization of the epoxy network, thereby enhancing mechanical properties such as the fracture toughness. The reduction in the free volume, along with the resulting denser crosslinked structure, plays a key role in the self-toughening effects observed [26].

## 4. Conclusions

This study explored a straightforward and effective self-toughening method to enhance the fracture toughness of high-T_g_ epoxy resins through the hybridization of structural isomeric curing agents, 3,3′- and 4,4′-DDS. By employing two curing agents with different reactivities in TGDDM, the curing behavior, thermal and mechanical properties, and network structure were examined based on their mixing ratios. The epoxy cured with a 7:3 mixture of 3,3′- and 4,4′-DDS (7*m*,3*p*-TGDDM) demonstrated the highest mechanical performance, achieving a 30% improvement in fracture toughness compared to *m*-TGDDM and a 100% improvement compared to *p*-TGDDM, without compromising its tensile properties. Crosslink density calculations based on the DMA results showed that the mixed curing agent system resulted in up to a 23% higher crosslink density compared to single curing agent systems. Solvent uptake measurements further revealed that 7*m*,3*p*-TGDDM had the smallest free volume size among all samples, attributed to the more complex and denser crosslink structure formed by the differing reactivities of the curing agents. This suggests that the use of mixed curing agents leads to a denser curing structure with a reduced free volume, thereby enhancing the mechanical properties. Moreover, the TGDDM cured with mixed curing agents maintained a high T_g_ of over 240 °C, ensuring excellent thermal stability alongside improved mechanical properties. These findings suggest that the combination of isomeric curing agents significantly alters the network formation mechanism, resulting in a denser cured epoxy structure with superior mechanical properties compared to systems using a single curing agent. Therefore, hybridizing structural isomeric curing agents provides an effective self-toughening method, enabling the development of high-performance epoxy resins with superior mechanical and thermal properties. Furthermore, it is expected that applying conventional thermoplastic-based toughening agents to the hybridized curing agent system developed in this study will yield even more pronounced toughening effects, resulting in highly toughened epoxy resins with high T_g_s.

## Figures and Tables

**Table 1:** Formulation of epoxy resin.

Epoxy Sample	Amount of Curing Agent (phr)	
	3,3′-DDS	4,4′-DDS
*m*-TGDDM	33.0	0
7*m*,3*p*-TGDDM	23.1	9.9
5*m*,5*p*-TGDDM	16.5	16.5
3*m*,7*p*-TGDDM	9.9	23.1
*p*-TGDDM	0	33.0

**Table 2:** Activation energy calculated by Kissinger and Ozawa methods.

Epoxy Sample	Activation Energy (kJ/mol)	
	Kissinger	Flynn–Wall–Ozawa (Average)
*m*-TGDDM	64.8	75.9
7*m*,3*p*-TGDDM	64.6	85.3
5*m*,5*p*-TGDDM	64.7	67.8
3*m*,7*p*-TGDDM	68.1	72.5
*p*-TGDDM	66.6	89.2

**Table 3:** Crosslink densities of the prepared epoxy samples.

Epoxy Sample	Storage Modulus (GPa)		T_g_ (°C)	Crosslink Density (mol/m^3^)
	25 °C	T_g_ + 20 °C		
*m*-TGDDM	9.9	0.124	241	9.28 × 10^3^
7*m*,3*p*-TGDDM	12.5	0.146	247	10.8 × 10^3^
5*m*,5*p*-TGDDM	12.6	0.138	253	10.1 × 10^3^
3*m*,7*p*-TGDDM	10.1	0.125	258	9.06 × 10^3^
*p*-TGDDM	10.0	0.122	266	8.77 × 10^3^

## Data Availability

The original contributions presented in this study are included in the article. Further inquiries can be directed to the corresponding authors.

## Supplementary Materials

The following supporting information can be downloaded at: https://www.mdpi.com/article/10.3390/polym17050695/s1.
