# Supplementary material for: Self-Toughened Epoxy Resin via Hybridization of Structural Isomeric Curing Agents
Source: Polymers (Basel). 2025 Mar 5;17(5):695. doi: 10.3390/polym17050695 (PMC11902468; doi:10.3390/polym17050695)
Supplement: Supplementary file 1 [file polymers-17-00695-s001.zip › polymers-3484471-supplementary.pdf]

polymers

MDPI

Supporting Information

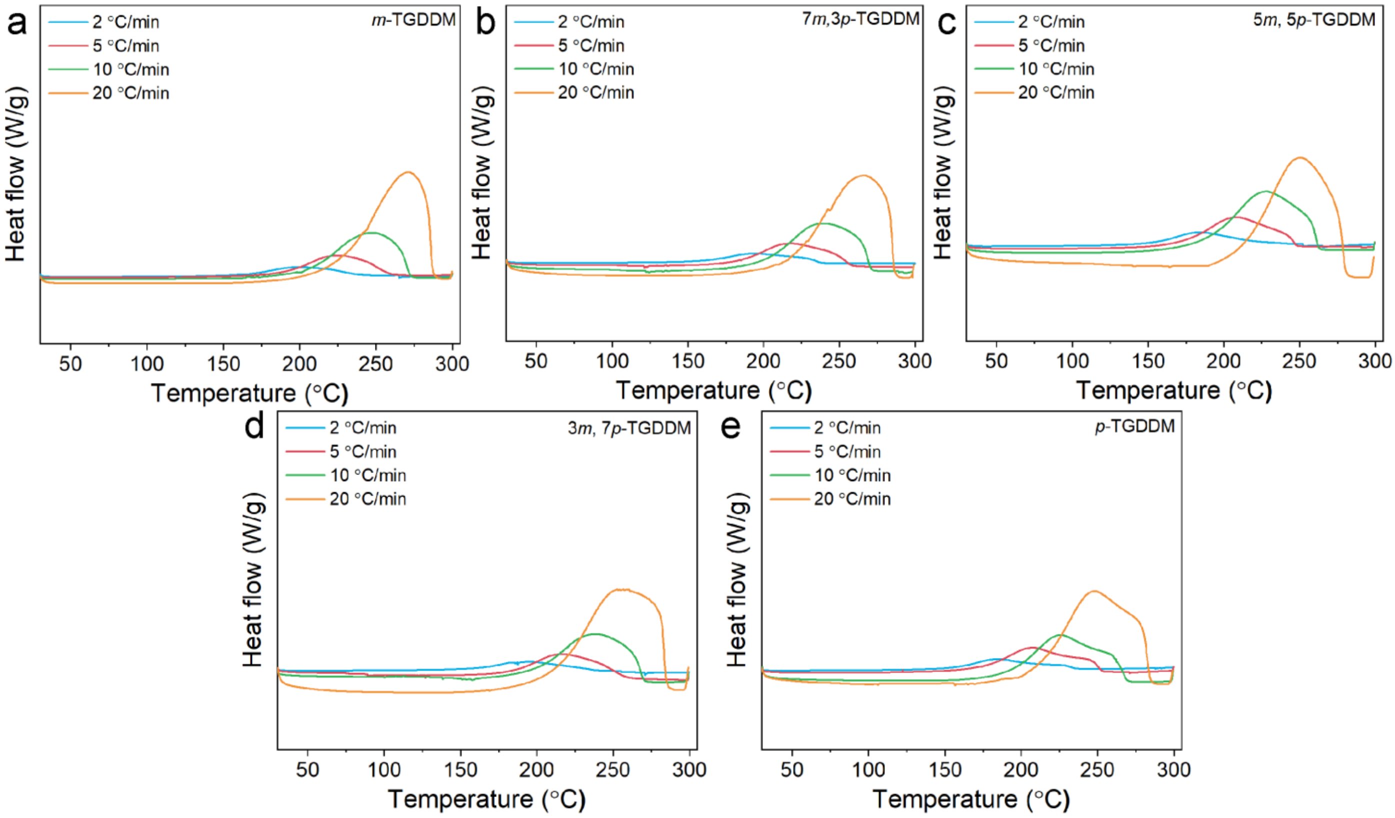

Figure S1. Heat flow from dynamic DSC of (a) *m*-TGDDM, (b) 7*m*,3*p*-TGDDM, (c) 5*m*,5*p*-TGDDM, (d) 3*m*,7*p*-TGDDM, and (e) *p*-TGDDM.

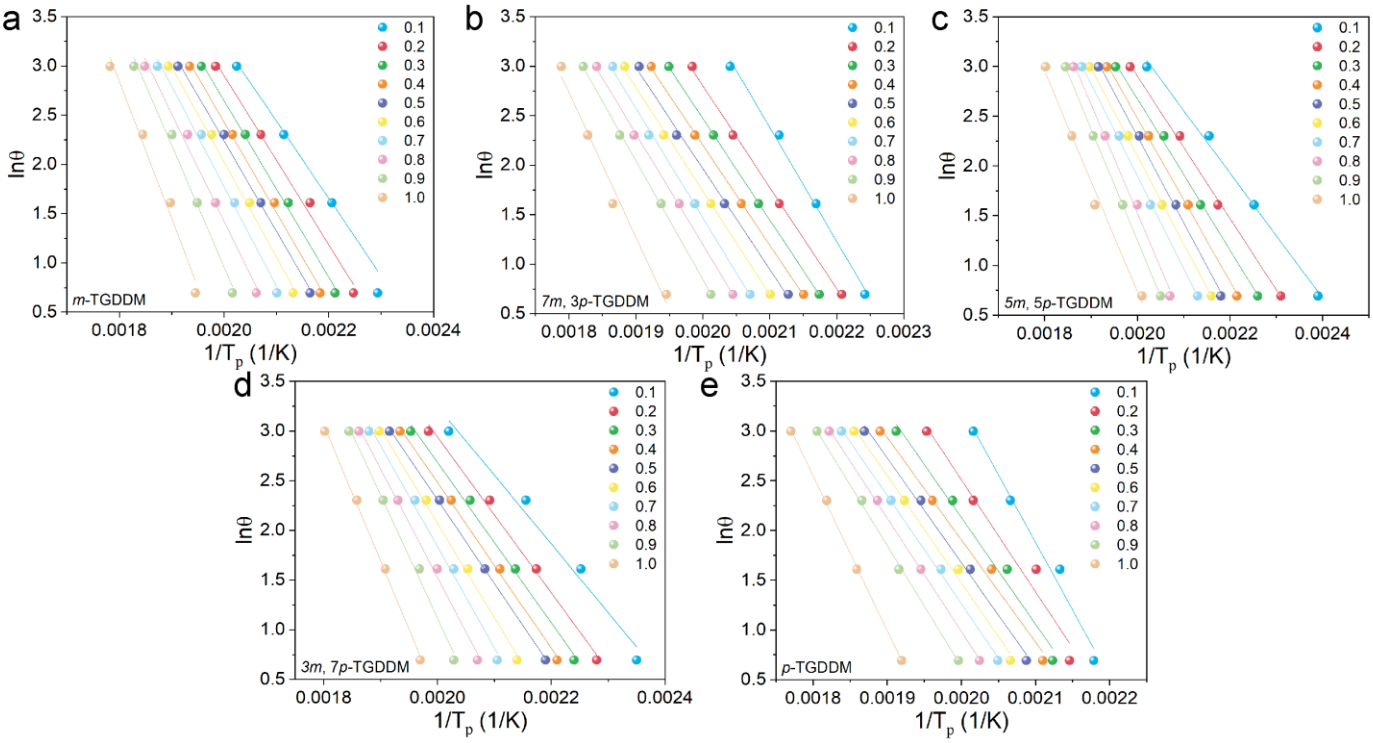

Figure S2. Flynn-Wall-Ozawa plots at different conersion rates; (a) *m*-TGDDM, (b) 7*m*, 3*p*-TGDDM, (c) 5*m*, 5*p*-TGDDM, (d) 3*m*, 7*p*-TGDDM, and (e) *p*-TGDDM.

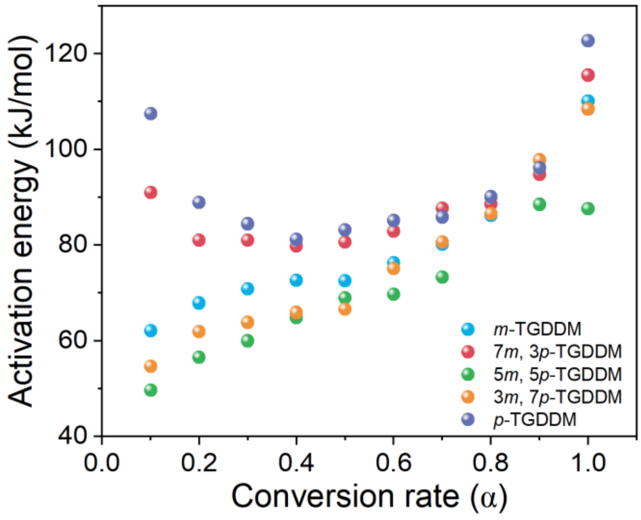

Figure S3. Activation energy calculated by Flynn-Wall-Ozawa method.

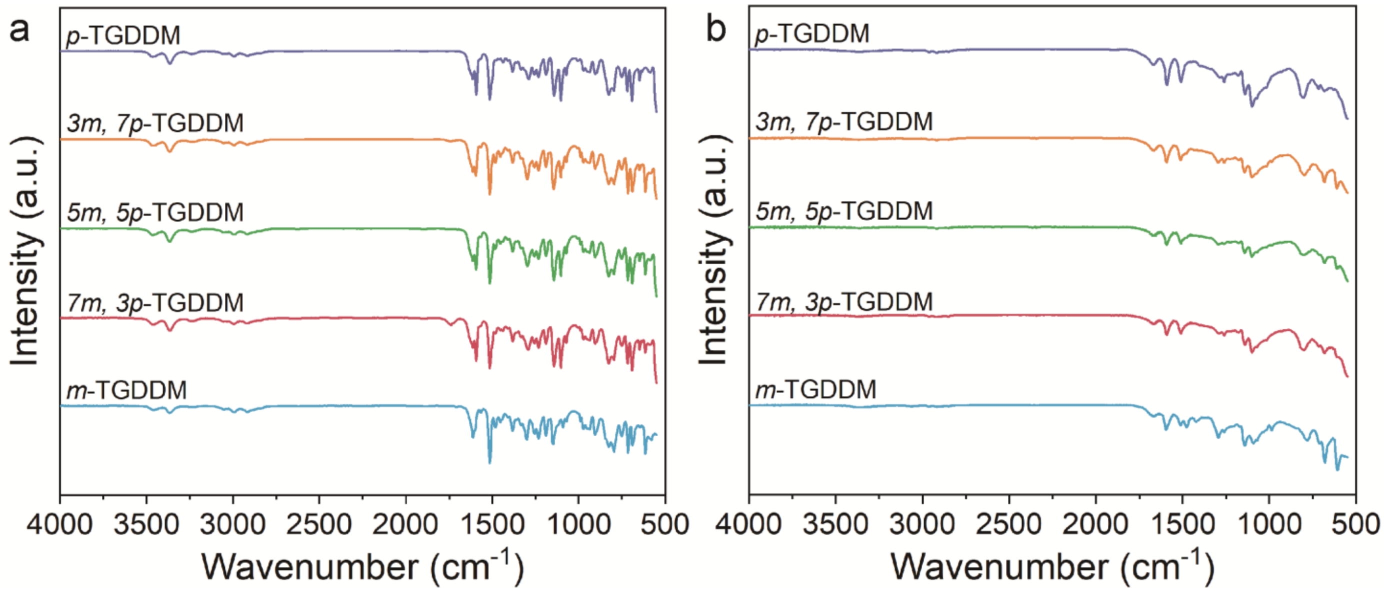

Figure S4. FT-IR spectra of the (a) uncured TGDDM and DDS mixtures and (b) cured epoxy resins.
